# Causes of changes in carotid intima-media thickness: a literature review

**DOI:** 10.1186/s12947-015-0041-4

**Published:** 2015-12-15

**Authors:** Baoge Qu, Tao Qu

**Affiliations:** Department of Gastroenterology, Taishan Hospital, Taian, Shandong 271000 P. R. China; Zhuhai Campus of Zunyi Medical College, Zhuhai, Guangdong 519041 P. R. China

**Keywords:** Ultrasound, Atherosclerosis, Peripheral vessel, Carotid intima-media thickness, Cardiovascular and cerebrovascular diseases, Risk factors

## Abstract

Atherosclerosis causes significant morbidity and mortality. Carotid intima-media thickness (CIMT) predicts future cardiovascular and ischaemic stroke incidence. CIMT, a measure of atherosclerotic disease, can be reliably determined in vivo by carotid ultrasound. In this review, we determined that CIMT is associated with traditional cardiovascular risk factors such as age, sex, race, smoking, alcohol consumption, habitual endurance exercise, blood pressure, dyslipidemia, dietary patterns, risk-lowering drug therapy, glycemia, hyperuricemia, obesity-related anthropometric parameters, obesity and obesity-related diseases. We also found that CIMT is associated with novel risk factors, including heredity, certain genotypic indices, anthropometric cardiovascular parameters, rheumatoid arthritis, immunological diseases, inflammatory cytokines, lipid peroxidation, anthropometric hemocyte parameters, infectious diseases, vitamin D, matrix metalloproteinases, and other novel factors and diseases. However, the conclusions are inconsonant; the underlying causes of these associations remain to be further explored.

## Background

Atherosclerosis is an important pathologic cause of cardiovascular (CV) and cerebrovascular diseases. Additionally, CV and cerebrovascular diseases are the leading causes of mortality in humans and can have significant impacts on morbidity. Therefore, the early prevention of CV and cerebrovascular diseases has become a focus of current research. Preclinical atherosclerosis has been related to higher coronary heart disease and stroke rates. Studies have demonstrated that carotid ultrasonography [1] is more sensitive than the coronary artery calcification score (CACS) for the detection of subclinical atherosclerosis. Hence, carotid intima-media thickness (CIMT) ultrasonography may represent an accessible and reliable method to detect subclinical atherosclerosis [2]. CIMT is significantly increased in patients with existing plaques [3], is a marker of subclinical organ damage and is an independent predictor of CV and cerebrovascular events. Several studies have reported that associations exist between CIMT and established risk factors. Thus far, the current concepts of the risk factors of CIMT in the existing literature are not unified. We combined the existing reports to summarize the risk factors of CIMT, including traditional risk factors that have been recognized, such as age, sex, smoking, alcohol consumption, blood pressure (BP), blood fat, blood sugar, lifestyle habits and others; however, the traditional risk factors do not explain all of the risks. A recent study revealed that >60Â % of CIMT cases were not explained by demographic and traditional CV risk factors, which highlights the need to study novel risk factors [4], which likely represent additional risk factors for carotid atherosclerosis. These novel risk factors refer to recently researched and not yet widely recognized risk factors, such as lifestyle, job pressure, certain diseases, genetic risk factors for certain diseases, and novel environmental, physiologic and pathologic parameters. Therefore, to further understand the factors that influence CIMT associated with traditional CV risk factors and novel risk factors, we have reviewed the relevant literature from PubMed by searching the keywords: "ultrasound" AND "carotid intima-media thickness" AND "clinical research" AND "traditional cardiovascular risk factors" OR "novel risk factors". Identifying novel risk factors associated with CIMT will be helpful for the prevention and treatment of early atherosclerosis.

## The associations between CIMT and traditional CV risk factors

### The associations between CIMT and age, sex, race, smoking, alcohol consumption and habitual endurance exercise

Age, sex, race, smoking and alcohol consumption play important roles in atherosclerosis. CIMT may be a valuable marker for CV risk in adults aged <45Â years who are not yet eligible for standard CV risk screening [5]. Gestational age was not found to affect CIMT [6], and age-related increases in CIMT correlated with a decline in cardiac diastolic function only in women [7]. CIMT was similar between healthy South Asian and European men [8]. Although males exhibited a significantly greater CIMT than females, the difference failed to reach statistical significance after adjusting for carotid diameter [9]. However, sex differences in the associations between childhood and adulthood risk factors and subclinical atherosclerosis were found [10]. Additionally, CIMT was positively associated with smoking [11] and alcohol consumption in men [12]. However, an opposite finding showed that alcohol consumption might also be beneficial for CIMT in men and that the effect of alcohol on CIMT might be mediated by lipid factors [13]. Habitual endurance exercises, particularly aerobic exercise, are important measures of atherosclerosis prevention. Previous studies have revealed that 8Â weeks [14] or 6 months [15] of aerobic exercise training might significantly improve the vascular structure and function in African Americans. The weight-adjusted maximal oxygen uptake capacity inversely correlates with CIMT [16]. However, although habitual endurance exercise might improve CV risk profiles, it does not reduce the magnitude of carotid atherosclerosis associated with age and CV risk factors [17]. Hence, determining the effects of different exercises on CIMT requires further studies.

### The associations between CIMT and BP

High BP has been recognized as an important risk factor for CV and cerebrovascular diseases. However, the associations between CIMT and different types of abnormal BP in different populations remain unknown. High BP is a major determinant of CIMT [18]. Systolic BP [19-21], diastolic BP (DBP) [10, 22], higher pulse pressure (PP) [6, 21], daytime BP [23], persistently elevated BP from childhood to adulthood [24], within-visit DBP variability in the normotensive population [25] and systolic BP variability (SBPV) [26] were also found to be positively associated with CIMT. SBP [27] appears to be a main pathological mechanism that indirectly affects CIMT through the hemodynamic pathway. This risk was reduced if BP that was elevated during childhood was resolved by adulthood [24]. Moreover, the correlation between BP variability (BPV) and left common carotid artery-intima-media thickness/number of plaques is stronger than right common carotid artery-intima-media thickness/number of plaques [26]. The increase in CIMT in Congolese hypertensive subjects was identified as a marker of arterial remodeling associated with a long history of uncontrolled hypertension rather than of early atherosclerosis [28]. In a sample of hypertensive African men, CIMT was negatively associated with glutathione (GSH) levels [29], suggesting that CIMT might contribute to the attenuation of GSH levels in the development of subclinical atherosclerosis. In summary, all types of hypertension are major causes of preclinical atherosclerosis. Thus, to achieve prevention of early atherosclerosis, proper measures should be taken to control hypertension.

### The associations between CIMT and dyslipidemia, dietary patterns and risk-lowering drug therapy

Healthy and unhealthy lifestyle changes during young adulthood are associated with decreased and increased risks, respectively, for subclinical atherosclerosis in middle age [30]. Dyslipidemia is an important factor for atherosclerosis that has been shown to be associated with CIMT [18]. Regression analysis demonstrated that the mean and maximum CIMT (CIMT[max]) are independently influenced by age, blood creatinine levels and non-high density lipoprotein (HDL) cholesterol levels [22]. In particular, patients with familial hypercholesterolemia (FH) are at increased risk of premature CV disease. Hypercholesterolemic children [31] and patients with FH [32] exhibited significantly higher CIMT. Results of CIMT meta-analysis strengthen the evidence of early atherosclerotic development in children with FH. Circulating low-density lipoprotein (LDL-C) concentrations were associated with CIMT [33, 34], and high levels of oxidized LDL (OxLDL) were significantly associated with progression and increased levels of CIMT [35-37]. Low levels of HDL cholesterol or relative levels of the HDL 3b subclasses and changes in the proportion of small HDL particles were significantly associated with an increased in CIMT [20, 38] and with the presence of carotid plaques [39]. Furthermore, among women, IMT(max) was significantly negative correlated to HDL cholesterol [40]. In the statin group, HDL cholesterol levels were associated with CIMT; in the combined therapy group, HDL cholesterol levels were the only significant correlate of CIMT [41]. Apoliproproteins are also important risk factors for atherosclerosis. Among them, apolipoprotein B(apoB) [33] and ApoB/ApoA1 [42] were significantly positively associated with CIMT, and the absence or very low levels of erythrocyte-bound apoB was associated with clinical and subclinical atherosclerosis [43]. In contrast, other studies revealed that ApoE genotypes and CIMT were not associated [44] and that ApoA1 has an inverse association with CIMT [42]. Therefore, whether changes in dietary patterns affect the risk of early atherosclerosis remains under debate. One study did not find an association between dietary patterns and IMT or plaques [45], and high versus low protein intake in infancy did not influence CIMT at 5Â years [44]. However, several studies have demonstrated that a Mediterranean diet has a protective effect on the CV system because lower adherence to a Mediterranean diet was shown to increase the risk of subclinical atherosclerosis [46]. Additionally, 12Â months of Mediterranean diet intervention caused a significant reduction in CIMT [31]. Interventions to lower LDL cholesterol from the suboptimal to the optimal range were shown to have potentially significant benefits to firefighters [34]. Atorvastatin [47], rosuvastatin [48] and fuvastatin [49] treatments slowed or reduced the progression of CIMT; intensive lipid lowering and antihypertensive therapy along with a reduction in central fat [50] may be considered a mandatory treatment strategy in young patients with FH to prevent atherosclerosis and to increase arterial stiffness. In men with CHD and high levels of Lp(a), atorvastatin use [51] results in an average 0.06Â mm decrease in CIMT over 6Â months. Adequate statin treatment [52] might delay carotid atherosclerosis in FH independent of Lp(a) levels. Hence, CIMT was associated with dyslipidemia and dysapoliproprotein but was not associated with protein intake. Therefore, intensive lipid-lowering therapy might be used to reduce the progression of CIMT in high-risk patients.

### The associations between CIMT and glycemia and hyperuricemia

Glycemia and hyperuricemia are also important risk factors for atherosclerosis-related CV and cerebrovascular diseases. The glycemic status is associated with all grades of carotid atherosclerosis, from early signs, as demonstrated by the IMT; to intermediate degrees, as demonstrated by the presence of carotid plaques; to advance atherosclerosis, as established by the presence of carotid stenosis [53]. Glycemia, including type 2 diabetes mellitus (T2DM) and impaired fasting glucose (IFG) [54], is a major independent determinant of CIMT in hyperglycemic patients. Insulin levels, the HOMA-IR index, total IGF-1 levels [55] and increased insulin resistance [56] are positively associated with CIMT; simultaneously, insulin resistance in obese adolescents [57] can impair changes in CIMT and can lead to the early development of atherosclerosis. Lower hemoglobin A1c levels [58] were also identified as a significant risk factor for carotid atherosclerosis in rural community-dwelling elderly Japanese men. Low levels of plasma obestatin might be related to early arteriosclerosis in patients with T2DM via increasing CIMT [59], considering that the elevated plasma obestatin levels might protect T2DM patients against carotid atherosclerosis to some extent. Despite reproducing the association between CIMT level and vascular risk in subjects with diabetes, no association between CIMT change and vascular risk was found [60]. However, in patients with T2DM, a 6-month intensive lifestyle modification intervention might result in improved glycemic control and decreased progression of CIMT [61]. Additionally, hyperuricemia is an independent risk factor for CV and cerebrovascular diseases. Hyperuricemia was inversely associated with subclinical carotid atherosclerosis in men; however, hyperuricemia-related renal impairment was a significant marker of subclinical carotid atherosclerosis in both men and women [62]. Therefore, a timely intervention for glycemia and hyperuricemia may slow the occurrence of early arteriosclerosis. This timeliness should be a primary focus for clinicians.

### The associations between CIMT, obesity-related anthropometric parameters, obesity and obesity-related diseases

Obesity-related anthropometric parameters, obesity and obesity-related diseases, including nonalcoholic fatty liver disease (NAFLD), alcoholic fatty liver disease (AFLD) and polycystic ovary syndrome (PCOS), have been reported to be associated with increased CV and cerebrovascular risks. However, the conclusions of the studies in the literature are inconsistent.

#### The associations between CIMT and obesity-related anthropometric parameters

Numerous obesity-related anthropometric parameters are used for evaluating the associations between obesity and atherosclerosis-related diseases. Studies have shown that higher CIMT correlated with obesity-related anthropometric parameters, including body mass index (BMI) [18, 63], waist circumference [18], waist-to-hip ratio (WHR) [39, 51] and WHtT [39]. Additionally, the associations between CIMT and fat distribution remain unknown. Visceral adipose tissue (VAT) thickness [64, 65], but not subcutaneous fat thickness [65], was associated with IMT in men and patients requiring peritoneal dialysis. Increased epicardial fat volume [66, 67] and peri-aortic root fat (PARF) [68] were shown to be independent risk factors of increased CIMT. However, a contradictory study [69] suggested that CIMT lacked a relationship with visceral obesity. Thus, prospective studies are needed to further determine the associations between CIMT and visceral obesity. Additionally, recent research has demonstrated that the mean CIMT varied across obesity phenotypes [59]; a higher CIMT value and positive associations with carotid plaque and carotid atherosclerosis were observed in only the metabolically abnormal obese subtype [70]. These contradictory findings [71] suggested that obesity does not affect vascular parameters related to early atherosclerosis, including CIMT in women with minor CV risk factors. Further research studies are warranted to identify the correlation between different fat distributions and CIMT.

#### The associations between CIMT and obese and obesity-related diseases

Recent studies have suggested that overweight, obese and obesity-related diseases, including NAFLD, AFLD and PCOS, are associated with CIMT. Overweight, obese and morbidly obese patients exhibit similar CIMT and small artery reactivity index values [72]. Patients with NAFLD [35, 73-75] and AFLD [35, 76] had significantly increased CIMT, and the association between NAFLD and subclinical atherosclerosis was independent of traditional risk factors [75]. ALD may also promote the premature increase in CIMT [76] via mechanisms that might include insulin resistance [77] and abnormal liver function, including ALT [78, 79] and GGT [78], in patients with NAFLD and NASH. Age and metabolic factors are also associated with CIM thickening in patients with ALD. PCOS is a common gynecological disease in women, and independent of obesity, patients with PCOS may present with increased CIMT as a direct result of androgen excess [80], suggesting that hyperandrogenism might increase atherosclerosis and CV risks. However, the findings of another study [71] did not support the above conclusions. Therefore, the precise relationships between CIMT and NAFLD, AFLD, and PCOS require further study.

## The associations between CIMT and novel risk factors

### The associations between CIMT and heredity and certain genotypic indices

Heredity and genetics play important roles in atherosclerosis. However, whether CIMT is associated with heredity and certain genotypic indices remains unknown. One study suggested that a different set of genes influences variation in CIMT and waist circumference [81]. Certain genetic loci that are determinants of human lung function also influence CIMT and CAD susceptibility [82]. The strongest association was reported between the rate of telomere shortening in adults between 53 and 60-64 years and CIMT in adults 60 to 64Â years of age [83]. However, the chromosome 9p21 locus does not influence CAD risk through a mechanism that also affects CIMT or that induces early changes in flow-mediated dilatation (FMD) [84]. The haptoglobin (Hp) 2-2 genotype is a significant predictor of premature atherosclerosis and is associated with increased CIMT in children with beta-thalassemia major [85]. Single-nucleotide polymorphisms (SNPs) in 7-dehydrocholesterol reductase/NAD synthetase-1 interacted with type 2 diabetes to significantly influence the progression of CIMT independent of 25(OH)D levels and established risk factors [86]. However, no significant associations were identified between the genotype at any of the SNPs and CIMT in 846 individuals with acceptable measurements [87]. Additionally, humans with the Osteopontin âˆ’66 TT genotype, particularly those without MetS, exhibit thicker CIM [88]. Hence, the associations between CIMT and heredity require further exploration to search for better etiological treatment.

### The associations between CIMT and anthropometric CV parameters

Recent studies have reported that certain cardiac- and artery-related changes may be associated with CIMT. These changes are important complications of T2DM. Studies have revealed that increased CIMT is associated with a high occurrence of cardiac autonomic neuropathy (CAN) [89], the level of coronary artery calcification (CAC) [90] and the coronary SYNTAX score [91]. However, coronary artery disease (CAD) variants were not found to be associated with CIMT and did not appear to mediate the risk of atherothrombosis through known risk factors [90]. Additionally, impaired FMD under various statuses and diseases might led to thicker CIM [78, 92, 93]. Their exact mechanism remains unknown and needs to be further studied.

### The associations between CIMT and rheumatoid arthritis (RA) and immunological diseases

Growing evidence has demonstrated that patients with RA, Behcet disease (BD), systemic lupus erythematosus (SLE), primary SjÃ¶gren syndrome, and psoriatic arthritis (PsA) have a higher risk for atherosclerosis and are associated with enhanced CV risk and subclinical vascular disease. RA is associated with an elevated risk of cardiovascular disease (CVD) [94] events and subclinical atherosclerosis [94, 95]. However, a contradictory study reported that RA does not result in CIM thickening [45]. Recent quantities clinical observations have confirmed that RA is associated with increased CIMT [96-99]. The mechanisms of RA-related CIM thickening included increased levels of Ox-LDL) [100, 101], vWF activity [102], serum mannose-binding lectin [103], and PWV [104] as well as increased levels of inflammation markers [99, 102] such as IL-17 [105], and CRP [106] and lower levels of Î²-carotene [101], vitamin D, CD34+ cells [100, 104] and NO [101]. Psoriasis is associated with increased mean CIMT [107] and may increase the burden of subclinical atherosclerosis [107, 106]. More severe subclinical atherosclerosis was observed in patients with PsA [108] compared with patients with cutaneous psoriasis without arthritis (PsC). The causes of atherosclerosis in PsA include markers of accelerated atherosclerosis such as ox-LDLs and NO [101] as well as endothelial dysfunction [106], PsA disease duration, more severe skin disease and increased inflammatory markers; however, atherosclerosis in PsA was not associated with traditional CV risk factors [108]. Furthermore, increased CIMT was associated with juvenile idiopathic arthritis [109], systemic lupus erythematosus [110], and BD [111]. Their mechanisms involved elevated myeloperoxidase levels in juvenile idiopathic arthritis [109], circulating levels of OxLDL and the % of LDL in systemic lupus erythematosus [110] and impaired FMD in BD [111]. Multivariate model analysis [112] showed that primary SjÃ¶gren syndrome (SS) might be an independent risk factor for arterial wall thickening when traditional risk factors for CVD were controlled. Another contradictory study [113] revealed no significant difference in CIMT between patients with primary SS and control patients. Additionally, a recent study found that euthyroid premenopausal women with autoimmune thyroiditis [114] and allergic predisposition in early childhood [115] might present significantly increased CIMT. Hence, rheumatoid immunological diseases were related to CIM thickening, and future prospective studies should account for the roles and mechanisms of the above diseases in the progression of atherosclerosis, and standard control and treatment of these diseases so that subclinical atherosclerosis can be prevented as soon as possible.

### The associations among CIMT and inflammatory cytokines, lipid peroxidation, anthropometric hemocyte parameters and infectious diseases

Inflammation and lipid peroxidation may play important roles in the development of atherosclerosis. However, whether immunological parameters, inflammatory cytokines, and lipid peroxidation influence CIMT remains unknown. Inflammatory cytokines such as CRP [43, 116] and lipid peroxidation levels [117] correlated with increases in CIMT. However, another study showed that the distributions of MnSOD, GSTM1 and GSTP1 genotypes according to CIMT, plaque type or plaque score did not significantly differ [118]. The findings of our recent study indicated that the increase in CIMT was not associated with cytokine profiles, oxidative balance or immune responses in patients with ALD [76]. Further research is needed to explore the potential associations between CIMT and inflammation and lipid peroxidation.

Leukocyte count and the neutrophil-to-lymphocyte ratio may be used as diagnostic and prognostic indicators of carotid atherosclerosis. Recent studies on anthropometric hemocyte parameters revealed that leukocytes play an independent role in early arterial damage and that these cells may reflect subclinical disease [119]. The neutrophil-to-lymphocyte ratio positively and moderately correlated with CIMT in an entire study population [120]. Because the above parameters are convenient and practical, they may be used to evaluate the possibility of infection-related CIM thickening.

The associations between CIMT and certain infectious diseases have been a focus of clinical studies. A preliminary study demonstrated that treated HIV infection is a risk factor for subclinical atherosclerosis in older individuals [42]. However, in a predominantly female HIV-infected population in South Africa [121], CIMT values were considerably high and were associated with CV risk factors rather than HIV-related factors. Another study supports this viewpoint: old age, longer protease inhibitor exposure, and impaired fasting glucose were independent factors associated with common CIMT in a population living with HIV [122]. Our previous study [123] indicated that either *H. pylori* infection with chronic alcohol use or chronic alcohol use alone could cause a significant increase in CIMT. Additionally, tooth loss and long-term periodontitis were related to subclinical atherosclerosis in men but not in women [124]. Because only a few studies have examined the association of inflammatory cytokines, lipid peroxidation, anthropometric hemocyte parameters and infectious diseases with CIMT, these parameters should be a focus of further studies with larger samples for verification of these findings.

### The associations among CIMT and socioeconomic position (SEP) and job stress

Few studies have shown that CIMT, SEP, and job stress are associated. However, these studies lack consistent conclusions. Present studies have revealed that job strain was related to increased CIMT in valine (Val)/Val carriers [125], adulthood [126] and its early nonsymptomatic stages in men [127]. Job strain appears to increase the risk of preclinical atherosclerosis. A catechol-O-methyltransferase genotype [125], a lack of leadership (a type A behavior component) [126], and an increase in hyperintense spots may explain the link between job strain and cerebrovascular disease [128]. Additionally, CIMT was associated with adult SEP [129] and shift work [130]. However, high-strain jobs and low job control were not associated with IMT independent of SEP; job stress did not explain the association between life course SEP and CIMT [129]. High job demands, interacting synergistically with low decision latitude, did not result in the development of carotid atherosclerosis in men or in women [131]. The mechanisms of these associations are unclear. Thus, we should strengthen the studies on associations between CIMT and SEP and job stress to take appropriate measures for early prevention and treatment of atherosclerosis.

### The associations between CIMT and vitamin D

Little is known regarding the association between vitamin D and CIMT. The studies regarding an association between serum vitamin D and CVD risk have presented inconsistent results. Recently, several studies have shown that low serum 25-hydroxyvitamin D3 (25(OH)D) levels in childhood were associated with increased CIMT in adulthood [132] and that subjects with serum 25(OH)D â‰¥20Â ng/mL had less mean-max CIMT progression following 3Â years of atorvastatin treatment, suggesting that underlying vitamin D deficiency may be involved in the response to atorvastatin in atherosclerosis prevention [133]. Hence, early application of 25(OH)D or coupling with oral lipid-lowering drugs such as statins may be helpful for preventing subclinical atherosclerosis. In contrast, serum levels of 25(OH)D were not independently associated with CVD risk factors [134] or CIMT [123, 135, 136]. However, increased serum 25(OH)D may predict subclinical atherosclerosis in nonsmokers [137]. Hence, the majority of the present studies do not support a protective effect of vitamin D against subclinical atherosclerosis. More studies are needed to explore whether 25(OH)D may affect the progression of atherosclerosis and the roles of 25(OH)D supplementation on the atherosclerotic process.

### The associations between CIMT and matrix metalloproteinases (MMPs)

Evidence suggests that MMPs may play an important role in atherosclerosis and be promising new therapeutic targets for treating subclinical atherosclerosis. Higher serum MMP-8 [37], MMP-9 [138, 139], TIMP-1 [138], MMP-9/TIMP-1 [138] and MMP-10 [140] levels were associated with increased CIMT. Hence, the above circulating MMP levels may be useful for identifying subclinical atherosclerosis. In summary, the roles of MMPs in atherosclerosis should be further examined in clinical studies for possible clinical application.

### The associations between CIMT and other novel factors and diseases

Recent studies have found that other novel factors and diseases such as hepatorenal function, microalbuminuria, low muscular strength, O3, beta-thalassemia minor, growth hormone deficiency and normal-tension glaucoma have certain relations to CIMT. Several studies have revealed that increased serum ALT levels (even high-normal levels) were associated with markers of CVD [51]. However, a study with opposing results reported that age-adjusted CIMT values did not differ according to GGT levels in males or females [53]. Microalbuminuria correlated with CIMT [141, 142], and muscular strength was inversely and independently associated with CIMT [92]. Childhood exposure to O3 may be a novel risk factor for CIMT in a healthy population of college students [143]. Additionally, higher CIMT was associated with the development of chronic kidney disease [144], growth hormone deficiency [145], beta-thalassemia minor [146], preeclampsia [142] and menopause transition [147]. However, recent studies could not show associations of elevated parathyroid hormone (PTH) concentrations [136] and normal-tension glaucoma [48] with altered CIMT. Therefore, the associations between CIMT and the above causes require further verification.

## Conclusions

This review of the recent literature presents preliminary evidence of certain associations between CIMT and traditional and novel CV risk factors, showing that CIMT is a good clinical predictor of early atherosclerosis, and that CIMT measurement is convenient, simple and practical. However, the majority of studies to date were retrospective, and the lack of prospective large-scale studies and of a unified measurement method used by clinical researchers limited our analysis. Determining which CIMT value (maximum CIMT, mean CIMT, minimum CIMT, left or right common carotid artery) is obviously associated with early atherosclerosis is necessary. These findings remind us that early prevention of atherosclerosis should start from infancy to enable detection of an abnormal state of health. Further studies focusing on both traditional and novel CV risk factors are needed. Because the human body is a unified whole, all types of abnormal states of health are likely to cause harm to the human body, including the blood vessel system. Hence, we should pay more attention to the health status of the human body, and to protect blood vessels and prevent early atherosclerosis, we should strengthen the treatment of abnormal states of health and diseases.

## Abbreviations

AFLD: Alcoholic fatty liver disease
Apo E: Apolipoprotein E
ALT: Alanine aminotransferase
baPWV: Brachial-ankle pulse wave velocity
CAC: Coronary artery calcification
CAD: Coronary artery disease
CAN: Cardiac autonomic neuropathy
CIMT: Carotid intima-media thickness
FMD: Flow-mediated dilation
GGT: Gamma-glutamyl transpeptidase
Hp: Haptoglobin
IFG: Impaired fasting glucose
LDL: Low density lipoprotein
LTL: Leucocyte telomere length
NALD: Non-alcoholic liver disease
PP: Higher pulse pressure
PCOS: Polycystic ovary syndrome
RA: Rheumatoid arthritis
sICAM-1: Soluble intercellular cell adhesion molecule-1
SNPs: Single nucleotide polymorphisms
T2DM: Type 2 diabetes
WHR: Waist-to-hip ratio
